# Distinct Microbial Community of Phyllosphere Associated with Five Tropical Plants on Yongxing Island, South China Sea

**DOI:** 10.3390/microorganisms7110525

**Published:** 2019-11-04

**Authors:** Lijun Bao, Wenyang Cai, Xiaofen Zhang, Jinhong Liu, Hao Chen, Yuansong Wei, Xiuxiu Jia, Zhihui Bai

**Affiliations:** 1 Research Center for Eco-Environment Sciences, Chinese Academy of Sciences, Beijing 100085, China; ljbao_st@rcees.ac.cn (L.B.); dretfr456@gmail.com (W.C.); chenhao@rcees.ac.cn (H.C.); yswei@rcees.ac.cn (Y.W.); 2 College of Resources and Environment, University of Chinese Academy of Science, Beijing 100049, China; 3 College of Biological Sciences and Biotechnology, Beijing Forestry University, Beijing 100083, China; 4 Institute of Naval Engineering Design & Research, Beijing 100070, China; fenny425@126.com (X.Z.); liujh690@sina.com (J.L.); 5 School of Environmental Science and Engineering, Hebei University of Science and Technology, Shijiazhuang 050018, China

**Keywords:** phyllosphere, tropical plants, bacteria, fungi, diazotrophs

## Abstract

The surfaces of a leaf are unique and wide habitats for a microbial community. These microorganisms play a key role in plant growth and adaptation to adverse conditions, such as producing growth factors to promote plant growth and inhibiting pathogens to protect host plants. The composition of microbial communities very greatly amongst different plant species, yet there is little data on the composition of the microbiome of the host plants on the coral island in the South China Sea. In this study, we investigated the abundances and members of a major microbial community (fungi, bacteria, and diazotrophs) on the leaves of five dominant plant species (*Ipomoea pes-caprae*, *Wedelia chinensis*, *Scaevola sericea*, *Cocos nucifera*, and *Sesuvium portulacastrum*) on the island using real-time quantitative polymerase chain reaction (PCR) and high-throughput amplicon sequencing. Quantitative PCR results showed that fungi and bacteria were ubiquitous and variable among different host plants. *Scaevola sericea* showed the lowest absolute abundance and highest diversity of fungi and bacteria, while *Cocos nucifera* had the lowest abundance and the highest diversity of diazotrophs compare to the other four plants. There was a small proportion of shared microorganisms among the five different plants, while unique fungi, bacteria and diazotrophs were significantly enriched for different host plant species in this study (*p* < 0.05). Some of the most abundant organisms found in the communities of these different host plants are involved in important biogeochemical cycles that can benefit their host, including carbon and nitrogen cycles.

## 1. Introduction

The leaf surface, also known as the phyllosphere, is a large and extremely diverse habitat for various groups of microorganisms, including many species of bacteria and fungi, and estimated to exceed 10^8^ km^2^ globally [1]. The phyllosphere is a unique and dynamic habitat and characterized by rapid changes in water and nutrient availability, dose of ultraviolet (UV) radiation and other environment stresses [2]. Previous studies have found that phyllosphere fungi and bacteria have adapted to the surfaces of leaf, interacting with plants positively by producing or modifying metabolites and hormones or interfering with pathogen growth, negatively by causing disease such as *Pseudomonas* and *Erwinia*, or neutrally [3,4,5,6,7]. The activities of microbial communities associated with the phyllosphere influence plant growth, health and the productivity of ecosystems [4,8], yet the plants also influenced the structure and function of microbial communities on the leaves [9]. The community structures of a phyllospheric microbiome were determined by a number of factors, which include host and microbial genotypes, interactions within microbiota and various abiotic factors [10].

Among the wide variety of microbiomes, bacteria have long been known to be the dominant member inhabiting plant leaves [4], often being found in numbers averaging 10^6^ to 10^7^ cells/cm^2^ of the leaf [11]. Molecular data indicate that hundreds of fungal species can be found even within a single plant [12]. Bacteria and fungi on the leaves play several functional roles in nutrient and carbon cycling through the fixation of nitrogen (N), nitrification, methanol degradation and so on, while the importance of these processes for the host plants and for the ecosystems they inhabit are still poorly understood [13,14,15]. Terhonen et al. [5] summarized that numerous studies on plant microbial communities resulted in the accumulation of a considerable amount of knowledge about the diversity of microorganisms (in particular, bacteria and fungi) associated with the model plants and their wild relatives, the important agricultural crops, and a number of tree species. At the same time, our knowledge of microorganisms associated with other plant species is still limited and often fragmentary, even in the case of the most common species of temperate and boreal forests [16].

Yongxing Island is the biggest coral island in South China Sea. *Ipomoea pes-caprae* (IP), *Wedelia chinensis* (WC), *Scaevola sericea* (SS), *Cocos nucifera* (CN), and *Sesuvium portulacastrum* (SP) are the common and abundant plants existed on the island. Among them, *Ipomoea pes-caprae* and *Wedelia chinensis* are medicinal plants, *Scaevola sericea* was mainly used to stabilize the eroding beaches, *Sesuvium portulacastrum* was widely used for marine ecological remediation, and *Cocos nucifera* is an extensively cultivated fruit tree. Owing to high humidity and temperature, the leaves of tropical plants typically differ from plant species in temperate to boreal ecosystems. Tropical plant phyllospheres can be very densely colonized by organisms [14]. Some previous studies examining microbial diversity in the phyllosphere found lower bacterial and fungal operational taxonomic unit (OTU) richness on temperate leaves than similar studies conducted in a tropical forest [17,18,19]. Some evidence has suggested that N_2_ fixation in the phyllosphere is the main mechanism for adding N in humid tropical ecosystems [20]. Cleveland [21] estimated that N_2_ fixation rates in tropical forests are commonly thought to be among the highest of any natural ecosystem, and indeed relatively high symbiotic rates have been suggested for a few tropical sites [22]. It is possible that targeted manipulation of microorganisms could lead to more environmentally and economically sustainable production systems. To accomplish the purpose, we collected leaves from five different host plants and utilized high-throughput amplicon sequencing to compare the major microbial community (including fungal, bacterial, and diazotrophic community) composition, structure, and interaction among different host plants phyllosphere and to elucidate the mechanism through which it influences the environment.

## 2. Materials and Methods

On 12 December 2017, we chose phyllosphere samples from five plants of *Ipomoea pes-caprae* (IP), *Wedelia chinensis* (WC), *Scaevola sericea* (SS), *Cocos nucifera* (CN), and *Sesuvium portulacastrum* (SP), which mainly grown on Yongxing Island, Hainan province (16°50′ N, 112°20′ E), in the South China Sea. This island has a true tropical maritime monsoon climate, with the annual average precipitation more than 1300 mm. The mean annual temperature is 26~27 °C, and peaks in temperature in May and June. Sampling occurred on a sunny day, and the week before sampling was sunny with an average temperature of 24 °C. Twelve individual plants of each plant species were haphazardly chosen. The sampled IP, WC, SS, and SP were not covered by any plants, including CN. Considering the heterogeneity of the tested leaves, leaf samples were collected from at least 4 plants to form a composite sample from 1.5 m × 1.5 m area, and three composite samples (more than 10 m apart) of phyllosphere were collected for each type of plant. In addition, the collecting area of CN was much bigger owing to the wide space between CN trees (>5 m).

Each leaf sample was cut with a pair of sterilized scissors and placed in a Labplas on ice. To standardize conditions as much as possible, we chose only green healthy-looking and intact leaves. All the phyllosphere samples were collected on a day in December 2017 in which they were all under identical weather conditions. The phyllosphere samples were quickly transported to the laboratory and immediately processed for DNA extraction. About each phyllosphere replicate, 30 g leaf samples were placed in 1000 mL sterile Erlenmeyer flask, which was then filled with 500 mL of sterile PBS buffer (pH 7.4, 1× phosphate-buffered saline buffer). To wash the microbial cells off the leaves, sonication (frequency 40 kHz) was performed for 6 min in an ultrasonic cleaning bath, shaking followed at 200 r min^−1^ for 20 min at 30 °C. Sonication (frequency 40 kHz) was then continued for a further 3 min. The cell suspension was filtered through a 0.22 µm × 50 mm sterile nylon membrane to separate microbial cells from the leaves. Phyllospheric DNA was directly extracted from each of the collected membranes. All DNA was extracted using a Fast^®^DNA SPIN Kit (MP Biomedicals, Santa Ana, CA, USA) according to manufacturer’s instructions. The DNAs sampleswere stored at −80 °C.

The quantitative PCR (qPCR) thermal profiles of the fungal internal transcribed spacer (ITS) region and bacterial *16S rRNA* gene were performed with primers ITS1/ITS2 and 799F/1115R, with the annealing temperatures of 55 °C and 57 °C, respectively (Table S1). Primer sets PolF/PolR were used to amplify the region of *nifH* gene, which is the DNA barcode marker for the molecular identification of diazotrophic bacteria [23] (Table S1). Quantitative PCR (qPCR) was performed using a CFX96 Optical Real-Time Detection System (Bio-Rad, Laboratories, Inc. Hercules, CA, USA) in a total volume of 20 µL containing 10.0 µL SYBR^®^Premix Ex Taq (Takara, Biotech, Dalian, China), 0.5 µM of each primer (10 mM), and 1 µL of the DNA template. To obtain the standard curve, a *nifH* gene fragment was cloned into the pMD19-T vector (Takara, Biotech, Dalian, China) and subsequently transformed into *Escherichia coli* JM109 competent cells. The plasmid DNA was extracted using a Plasmid Purification Kit (Takara, Biotech, Dalian, China). The plasmids containing the correct fragment length were selected and verified. These were then used as a template to generate a standard curve. Blanks were run with sterile water as the template instead of the DNA. Specific target gene amplification was confirmed by agarose gel electrophoresis and melting curve analysis. Each DNA sample was determined in triplicate, and amplification efficiencies ranged between 90.1–102.5% with an R^2^ value of 0.991. The cycling conditions for the three genes were as follows: 40 cycles of 30 s at 95 °C, annealing for 30s at the temperatures in Table S1, and extension at 72 °C for 30 s, and a final extension at 72 °C for 8 min.

Primers ITS1/ITS2, 799F/1115R, and PolF/PolR were used to amplify the region of the ITS1, *16S rRNA*, and *nifH* gene, respectively. PCR was performed in 50 µL reaction volumes containing 25 µL of Premix Taq DNA polymerase, 0.5 µL of the forward primer (20 mM), 0.5 µL of the reverse primer (20 mM), 23 µL of double distilled water (ddH_2_O), and 1 µL of the DNA template (20 ng total DNA). Illumina libraries were constructed using the MiSeq Reagent Kit v3 according to manufacturer’s instructions. High-throughput, paired-end sequencing was performed on the Illumina MiSeq PE250 platform. Bacteria, fungi, and nitrogen-fixing bacteria sequencing data were deposited in the SequenceShort Read Archive (SRA) at the National Center for biotechnology information (NCBI) with accession numbers SRP148402, SRP158738, and SRP144329, respectively.

Sequencing data analyses were performed using the free online platform of Majorbio I-Sanger Cloud Platform (Available online: www.i-sanger.com, Shanghai, China). To calculate community similarities, an OTU-based hierarchical cluster analysis with the unweighted pair group method of arithmetic means, a principal co-ordinates analysis (PCoA) based on Bray-Curtis distance matrices, as well as an analysis of similarity (ANOSIM), were carried out using the vegan package of the R software (Version 3.1.2). Cladograms were drawn using the Huttenhower Galaxy web application (Huttenhower Lab, Boston, MA, USA) with the LEfSe algorithm (Available online: http://huttenhower.sph.harvard.edu/galaxy). Linear discriminant analysis (LDA) effect size was used to elucidate differences in microbial taxa [24]. An LDA score of ≥2 indicated an important contribution to the model. Using the *16S rRNA* gene data, the metagenome of the phyllospheric communities was predicted by Tax4Fun tools, which is connected to the SILVA database [25]. The Kyoto Encyclopedia of Genes and Genomes (KEGG) database was selected and used to predicted molecular functions. Significant differences between the absolute abundance and diversity of fungi, bacteria and diazotrophs were determined by one-way analysis of variance (ANOVA) analysis using SPSS 16. The statistical significance level of all the analysis was 0.05.

## 3. Results

### 3.1. Quantitative Abundance

**Figure 1:**
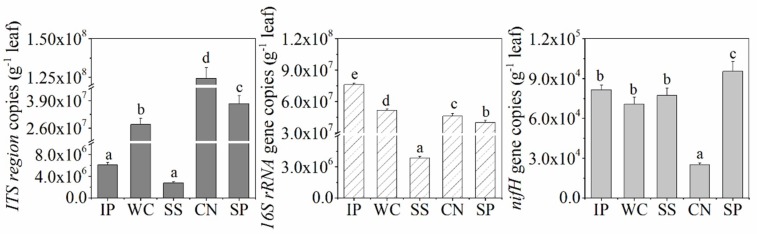
Internal transcribed spacer (ITS) region, *16S rRNA* gene and *nifH* gene copy number as quantified by real-time polymerase chain reaction (PCR) in the phyllosphere samples. Different lowercase letters (a, b, c, d, e) above the columns indicate significant differences among phyllosphere samples at *p* < 0.05. IP indicates *Ipomoea pes-caprae*, WC indicates *Wedelia chinensis*, SS indicates *Scaevola sericea,* CN indicates *Cocos nucifera,* SP indicates *Sesuvium portulacastrum*.

The abundance of bacteria, fungi, and diazotrophs among the phyllosphere samples of IP, WC, SS, CN and SP were detected by performing qPCR assays and found to be different (Figure 1). The qPCR data showed sample-specific gene copy numbers for fungi (6.08 × 10^6^ ~ 1.24 × 10^8^ copies per g leaf) and bacteria (3.84 × 10^6^–7.60 × 10^7^ copies per g leaf). The abundance of diazotrophs with *nifH* gene in phyllosphere ranged from 2.50 × 10^4^ to 9.54 × 10^4^ copies per g leaf. Among the five different phyllosphere samples, CN had the lowest *nifH* gene abundance and highest copies of ITS region, in contrast, the abundance of *nifH* gene in SP was the highest, and SS had the extremely low abundance about fungi and bacteria (*p* < 0.05). In addition, the abundance of bacteria in IP was the higest (*p* < 0.05).

### 3.2. Alpha Diversity

After quality filtering, the data obtained 340,835 high-quality ITS1 amplicon reads with 52,727 to 70,326 sequences per sample and 340,835 high-quality *16S rRNA* gene sequences with 22,438 to 55,137 sequences per sample, and the numbers of OTUs (97% similarity) ranged from 71 to 448 and from 304 to 1780, respectively, depending on Table S2. Alpha-diversity of fungi and bacteria was both determined by the Shannon diversity index, Chao1 richness index and the Heip’s evenness index, calculated with 52,727 and 22,438 rarefied sequences per sample, respectively. Good’s coverage was determined to estimate sampling completeness [26]. Good’s coverage of fungi and bacteria ranged from 0.9985 to 0.9994 and 0.9862 to 0.9968 (both at the 97% similarity level), respectively, which indicated that the sequencing depth contained almost all fungi and bacteria communities in phyllosphere samples. Results showed that the fungal and bacterial Shannon index, Chao1, and Heip’s evenness index of phyllosphere SS were the highest compared with the other four plant species, while the lowest Shannon index, Chao1, and Heip’s evenness index of fungi and bacteria were detected at SP and IP, respectively (*p* < 0.05). In addition, CN and WC also had the lowest Heip’s evenness index of fungi, and SP had the lowest Heip’s evenness index of bacteria (Table 1).

We also examined the diazotrophic alpha-diversity in the tropical phyllosphere. After quality filtering, 340,835 high-quality sequences with 11,314 to 19,537 sequences per sample were obtained, and the numbers of OTUs (97% similarity) ranged from 104 to 480, depending on the Table S2. Diazotrophic alpha-diversity was also determined by the Shannon diversity index, Chao1 richness index and the Heip’s evenness index, both calculated with 11,314 rarefied sequences per sample [27,28]. Good’s coverage was determined to estimate sampling completeness [26]. Good’s coverage ranged from 0.9980 to 0.9992 (at the 97% similarity level), indicating that this sequening depth contained almost all diazotrophic communities. The highest Shannon index and Chaol value was observed for CN, while the Heip’s index is significantly lower than that of WC. In a comparison of forementioned five phyllosphere samples, the Shannon diversity index, Chao1 index and Heip’s index of IP were totally lower than that of the other four phyllosphere samples, which was consistent with the results of minimum bacterial diversity of IP (Table 1).

Given the above, of all determined samples, the phyllosphere of CN showed the highest diazotrophic richness and evenness. Phyllosphere of IP showed the lowest diazotrophic and bacterial richness and evenness compared to other phyllosphere samples. Phyllosphere of SP harbored the lowest fungal richness and evenness, while the phyllosphere of SS both contained the highest fungal and bacterial richness and evenness.

### 3.3. Beta Diversity

**Figure 2:**
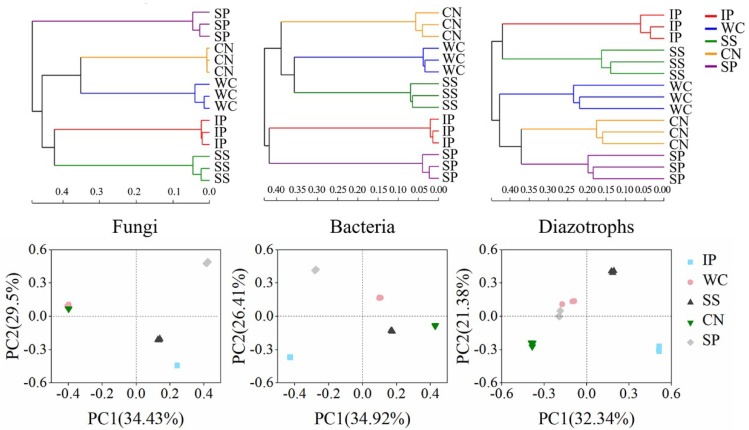
Hierarchical cluster analysis and principal coordinates analysis (PCoA) of fungi, bacteria and nitrogen-fixing bacteria community composition in sampled phyllosphere. IP indicates *Ipomoea pes-caprae*, WC indicates *Wedelia chinensis*, SS indicates *Scaevola sericea,* CN indicates *Cocos nucifera,* SP indicates *Sesuvium portulacastrum*.

To compare the similarities and differences among phyllosphere microorganism compositions, including fungal, bacterial and diazotrophic community, we used hierarchical cluster analysis, principal coordinates analysis (PCoA) and ANOSIM, based on the OTU composition. These analyses showed that the community compositions of fungi, bacteria and diazotrophs amongst the five tropical phyllosphere were clearly distinct, respectively (Figure 2). The hierarchical cluster analysis showed three groups about fungi community composition, as follows: Phyllosphere samples of IP, SS; phyllosphere samples of WC, CN and phyllosphere samples of SP. The bacterial community composition analysed by hierarchical cluster displayed three groups, phyllosphere samples of IP and SP; phyllosphere samples of SS and WC; and phyllosphere samples of CN. The diazotrophic community composition analysed by hierarchical cluster displayed three groups, phyllosphere samples of IP and SS; phyllosphere samples of SP and CN; and phyllosphere samples of WC. The PCoA analysis obtained a similar result. The dissimilarities between community compositions were calculated using an ANOSIM, which showed significant differences among phyllosphere versus phyllosphere (*p* < 0.01, Tables S3–S5). This indicated that plant species influenced the phyllosphere fungal and bacterial community structure, including diazotrophic community structure and the sample-specific taxonomic distribution of fungi, bacteria and nitrogen-fixing bacteria found between different plant species.

### 3.4. Sequence Variants and Dominant Genera

To acquire comprehensive knowledge of the microorganism community in phyllosphere, we analyzed the composition of total fungi and bacteria, via ITS1 and *16S rRNA* gene sequencing, respectively.

#### 3.4.1. Fungal Sequences

**Figure 3:**
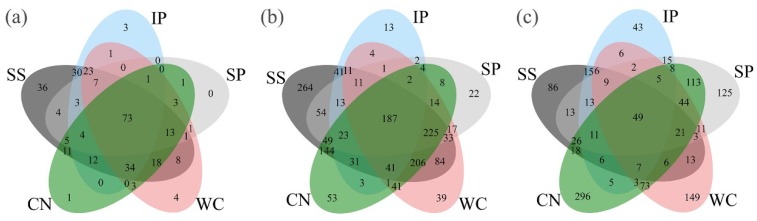
Venn diagrams showing the distribution of fungal (**a**), bacterial (**b**) and diazotrophic (**c**) operational taxonomic units (OTUs) between phyllosphere samples. IP indicates *Ipomoea pes-caprae*, WC indicates *Wedelia chinensis*, SS indicates *Scaevola sericea*, CN indicates *Cocos nucifera*, SP indicates *Sesuvium portulacastrum*.

**Figure 4:**
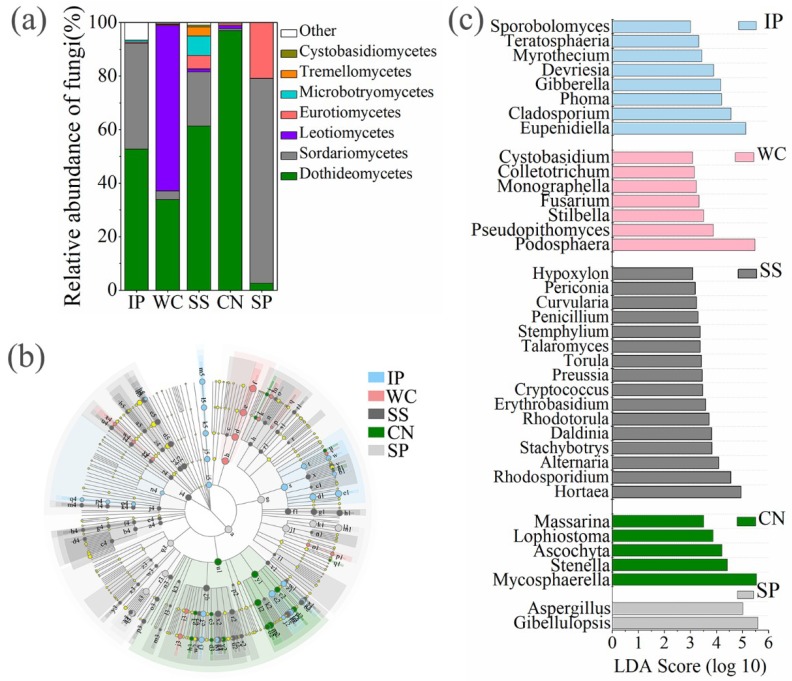
The relative abundances of fungi at class level of five different phyllosphere samples (**a**). Taxonomic differences among different phyllosphere samples by a linear discriminant analysis (LDA) coupled with effect size (LEfSe). Taxonomic representation of statistically and biologically consistent differences among different phyllosphere samples (**b**). LDA scores were calculated by the LDA effect size, using the linear discriminant analysis to assess the effect size for each differential genera with relative abundance of the taxa comprising more than 0.1% of the fungal sequences in each sample (**c**). IP indicates *Ipomoea pes-caprae*, WC indicates *Wedelia chinensis*, SS indicates *Scaevola sericea*, CN indicates *Cocos nucifera*, SP indicates *Sesuvium portulacastrum*.

High-quality ITS1 reads were clustered in to 688 non-singleton operational taxonomic units (OTUs), with a large majority being assigned to the phylum Ascomycota (96.06% of all reads), and Basidiomycota accounted for 2.72% (Figure S1). Among all detected fungal sequences, a total of 73 OTUs, accounting for 10.61% of the total OTUs were shared between the phyllosphere samples from five different plant species (Figure 3a). At the class level (Figure 4a), phyllosphere samples of IP, WC, SS and CN were mostly composed of Dothideomycetes with the relative abundance 52.67%, 33.77%, 61.3% and 96.96%, respectively, while phyllosphere of WC had a large proportions of Leotiomycetes (61.79%), which more abundant than Dothideomycetes (33.77%). In addition, IP and SS contained Sordariomycetes with relative abundance of about 39.59% and 20.28%, respectively. The fungal profiles of IP was similar to the SS, while fungal profiles of WC was similar to CN (Table S3, Figure 4a). Interestingly, the fungal community distribution was significantly different between phyllosphere sample SP and the other four phyllosphere samples, as well as the fungal community structure. SP had a greater relative abundance of Sordariomycetes (76.5%) and Eurotiomycetes (20.83%), in comparison to a low abundance (~2.59% of all reads) of Dothideomycetes.

The composition of enrichment taxa varied greatly amongst different host plant species (Figure 4b). At genus level, there are 38 genera with relative abundance of the taxa comprising more than 0.1% of the fungal sequences in each sample, which were significantly (*p* < 0.05) dominant among phyllosphere samples of different plant species, respectively (Figure 4c). *Eupenidiella*, *Cladosporium, Phoma*, and *Gibberella* were the dominant genera with a relative abundance as high as 41.57% of all reads in the IP, the dominant taxa in WC sample comprised of *Podosphaera* with a relative abundance of 61.75%, *Hortaea*, *Rhodosporidium*, and *Alternaria* were the main genera with a relative abundance as high as 26.57% of all reads in the SS, *Mycosphaerella* was the most abundant genus in CN with a relative abundance of 71.75%, instead, a relative high abundance of *Aspergillus* and *Gibellulopsis* (96.53%) was detected in the SP (Figure 4c). Moreover, 31 shared genera detected both at five phyllosphere samples, which occupied 80.02%~99.78% of the corresponding phyllosphere samples (Figure S2).

#### 3.4.2. Bacterial Sequences

**Figure 5:**
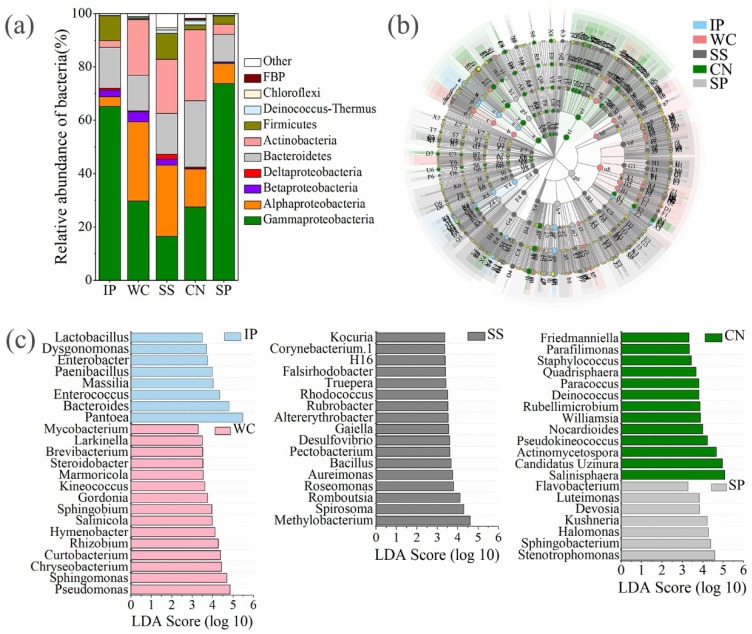
The relative abundances of bacteria at phylum/class level between different phyllosphere samples (**a**). Taxonomic differences among different phyllosphere samples by a linear discriminant analysis (LDA) coupled with effect size (LEfSe). Taxonomic representation of statistically and biologically consistent differences among different phyllosphere samples (**b**). LDA scores were calculated by the LDA effect size, using the linear discriminant analysis to assess the effect size for each differential genera with relative abundance of the taxa comprising more than 0.5% of the bacterial sequences in each sample (**c**). IP indicates *Ipomoea pes-caprae*, WC indicates *Wedelia chinensis*, SS indicates *Scaevola sericea*, CN indicates *Cocos nucifera*, SP indicates *Sesuvium portulacastrum*.

Taxonomic classification revealed that high-quality *16S rRNA* gene reads were clustered in to 2895 non-singleton OTUs, and the presence of numerically dominant phylotypes of Gammaproteobacteria, Alphaproteobacteria, Betaproteobacteria, Deltaproteobacteria, Bacteroidetes, Actinbacteria, Firmicutes, Deinococcus-Thermus, Chloroflexi and FBP both in phyllosphere samples, which the sum is more than 95% about the total bacteria (Figure 5a). A total of 187 OTUs, accounting for 6.46% in all bacterial OTUs were shared between five different phyllosphere samples (Figure 3b).

The bacterial community distribution of WC was more similar to SS and CN than IP and SP (Table S4, Figure 5a). In phyllosphere samples of IP and SP, the largest group consisted of Gammaproteobacteria accounting for 65.20% and 73.72%, respectively, and then Bacteroidetes (15.26% and 10.31%, respectively). The bacterial community in the phyllosphere samples of WC, SS and CN were mainly composed of Gammaproteobacteria, Alphaproteobacteria, as well as Bacteroidetes, Actinbacteria. Members of Gammaproteobacteria were found in all the detected phyllosphere samples with relatively high abundance, while the distribution of dominant genera between five phyllosphere samples differed greatly (Figure 5b). At genus level, there are 60 genera with relative abundance of the taxa comprising more than 0.5% of the bacterial sequences in each sample, which were significantly (*p* < 0.05) dominanted among phyllosphere samples of different plant species, respectively (Figure 5c). *Pantoea* (59.38%) and *Bacteroides* (12.76%) were dominant in IP, and *Pseudomonas*, *Sphingomonas*, *Chryseobacterium*, *Curtobacterium*, and *Rhizobium* were the dominant genera with a relative abundance as high as 42.22% of all reads in WC, in contrast *Methylobacterium*, *Spirosoma* and *Romboutsia* were dominant in SS with a relative abundance of 15.20%. *Salinisphaera*, *Candidatus Uzinura* and *Actinomycetospora* were the dominant genera in CN with a relative abundance of 51.18%, instead, *Stenotrophomonas*, *Sphingobacterium*, *Halomonas*, and *Kushneri* were the dominant genera with a relative abundance as high as 20.62% of all reads in the phyllosphere SP (Figure 5c). In addtion, 33 shared genera (relative abundance of the taxa comprising more than 0.1% of the bactrial sequences in each sample) detected both at the five phyllosphere samples, which occupied 77.73%, 73.53%, 51.91%, 35.74%, and 81.00% of all reads in IP, WC, SS, CN, and SP, respectively (Figure S3).

#### 3.4.3. Diazotrophic Sequences

**Figure 6:**
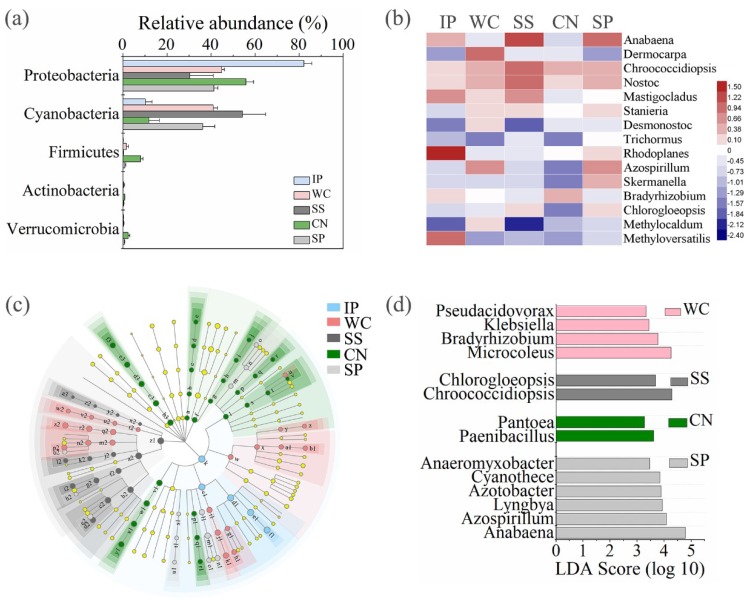
The relative abundances of diazotrophs at phylum level of five different phyllosphere samples (**a**). Heatmap of shared diazotrophic genera between the five phyllosphere samples (**b**). Taxonomic differences among different phyllosphere samples by a linear discriminant analysis (LDA) coupled with effect size (LEfSe). Taxonomic representation of statistically and biologically consistent differences among different phyllosphere samples (**c**). LDA scores were calculated by the LDA effect size, using the linear discriminant analysis to assess the effect size for each differential genera with relative abundance of the taxa comprising more than 0.1% of the diazotrophic sequences in each sample (**d**). IP indicates *Ipomoea pes-caprae*, WC indicates *Wedelia chinensis*, SS indicates *Scaevola sericea*, CN indicates *Cocos nucifera*, SP indicates *Sesuvium portulacastrum*.

High-quality *nifH* gene reads were clustered in to 1,199 non-singleton OTUs, with a large majority being assigned to the phylum Proteobacteria as well as Cyanobacteria (Figure 6a). Among all the detected diazotrophic sequences, a total of 49 OTUs, accounting for 4.09% of the total OTUs were shared between all the phyllosphere samples from five different plant species (Figure 3c). In addition, 15 shared genera (with relative abundance of the taxa comprising more than 1.0% of the diazotrophic sequences in each sample) were detected among the five different phyllosphere (Figure 6b), which accounted for 87.62%, 34.22%, 50.69%, 13.15%, and 42.39% of all sequencing reads of IP, WC, SS, CN, and SP respectively. While the abundance of diazotrophic community composition was different between the five type phyllosphere samples. At phylum level, IP and CN had a greater relative abundance of Proteobacteria of 82.12% and 55.76%, respectively, which was strictly higher than Cyanobacteria (the relative abundance of 10.11% and 11.65%, respectively). In contrast, the relative abundance of Cyanobacteria of SS accounting for 54.18% was higher than Proteobacteria of 30.27%. Instead, WC and SP had no obvious difference between the abundance of Proteobacteria and Cyanobacteria (Figure 6a).

Meanwhile, we found that the composition of enrichment diazotrophic genera varied greatly amongst different host plant species (Figure 6c). At genus level, there are 14 genera with relative abundance of the taxa comprising more than 0.1% of the diazotrophic sequences in each sample, which were significantly (*p* < 0.05) dominant among phyllosphere samples of different plant species, respectively (Figure 6d). Comparing all the phyllosphere samples, *Microcoleus* belonged to Cyanobacteria and *Bradyrhizobium* belonged to Alphaproteobacteria were the dominant genus in WC with the abundance of 9.75%, in comparison with other four phyllosphere samples. Instead, *Chroococcidiopsis* belonged to Cyanobacteria and *Chlorogloeopsis* belonged to Alphaproteobacteria as the dominant genera in SS with the abundance of 12.13%. *Paenibacillus* belonged to Firmicutes and *Pantoea* belonged to Gammaproteobacteria were dominant in CN with the abundance of 2.23%. *Anabaena* belonged to Cyanobacteria and *Azospirillum* belonged to Alphaproteobacteria as the dominant genera in SP with the abundance of 22.10%.

### 3.5. Predictive Metagenome Analysis

Using Tax4Fun tools as a predictive exploratory tool, we found that overall 45 level 2 orthology groups (KOs in KEGG) were observed in phyllospheric bacterial community (Figure S6). Almost half of the major functions were classified into multiple metabolism groups, including amino acid metabolism, carbohydrate metabolism, energy metabolism, and glycan biosynthesis and metabolism. Although the bacterial composition was quite different between different plant species, their functional classification appeared to be more consistent with most basic metabolic pathways being similar. Except the relative abundance of membrane transport in CN was strictly lower than that in the other plant species. Several different pathways were found (Figure S7, data set 2). Function prediction showed some differences in biosynthesis and metabolism groups. For example, the relative abundance of lipid biosynthesis proteins was significantly different between five types of plants, and CN had the highest relative abundances than the other four plant species.

## 4. Discussion

Microbial communities colonize plant surfaces, enabling interactions that are closely related to plant growth and health and play an important role of carbon and nitrogen cycling in the terrestrial ecosystem [4,14,29]. In this study, we found the abundance, diversity and composition of fungal and bacterial community structures of the phyllosphere of *Ipomoea pes-caprae*, *Wedelia chinensis*, *Scaevola sericea*, *Cocos nucifera* and *Sesuvium portulacastrum* varied greatly, which is associated with the differences of morphology and physiology of different plant species, and in turn likely related to the phylogenetic distance of the five plant species, where *Ipomoea pes-caprae* belongs to the family Convolvulaceae, *Wedelia chinensis* belongs to the family Compositae, *Scaevola sericea* belongs to the family Goodeniaceae, *Cocos nucifera* belongs to the family Palmae and *Sesuvium portulacastrum* belongs to the family Aizoaceae.

### 4.1. Quantitative Abundance and Diversity of Microbial Communities among Five Plant Species

On the whole, the abundance and diversity of microbiome in the five phyllosphere samples was significantly different. SS had the lowest abundance of fungal and bacterial population sizes and this might be related to the waxy leaves known as features of SS [30], which are not suitable for colonization of large numbers of microorganisms. Some cultivation studies found that bacteria are more abundantly on succulent herbaceous plants than on grasses or waxy broad-leaved plants [9,31]. Lindow et al. [4] concluded that plant species appeared to influence the microbial carrying capacity of the leaf. Redford et al. [17] suggested a significant correlation between host plant species and bacterial community phylogeny. Such differences in the abundance and diversity of microbial communities among different plant species can be explained by different physical characteristics of the leaf cuticles [32], nutrient concentrations [2,33], or by the different formations of secondary plant metabolites [34].

### 4.2. Fungal Communities among Five Plant Species

On the phylum level for fungal taxonomic composition, the primarily character of the phyllosphere community in the tropics did not differ greatly from temperate or tropical plants, including relatively high abundances of the phylum Ascomycota, as generally observed from previous studies [19,35]. Dothidiomycetes, Sordariomycetes, Leotiomycetes and Eurotiomycetes belonging to the phylum Ascomycota were both found in five plant species in this study, Yao et al. [35] found endophytic and epiphytic were dominated by Dothideomycetes and Tremellomycetes in a tropical mangrove ecosystem. Some members, like *Alternaria*, *Aspergillus*, *Penicillium*, *Fusarium*, are considered as endophytes supported by various investigations [36,37], which were both detected in the five plant species of this study. This phenomenon further emphasizes that there is an overlap between epiphytic and endophytic fungal communities [38]. Dothidiomycetes is one of the largest classes and also has the most ecologically diverse, with life histories that include pathogens, saprotrophs, and lichenized fungi [39]. Most of the dominant fungi, like *Mycosphaerella*, *Eupenidiella*, *Hortaea*, *Cladosporium*, *Ascochyta*, *Phoma*, *Alternaria*, *Devriesia*, *Pseudopithomyces*, and *Pyrenochaeta* within the Dothidiomycetes, and *Gibellulopsis*, and *Nigrospora* within the Sordariomycetes, and *Podosphaera* within Leotiomycetes, and *Aspergillus* within Eurotiomycetes, were known as opportunistic pathogens, and their abundance were significantly different among the five plants, indicating that these pathogenic fungi are in particular associated with host plant species and we should pay more attention to the health of these coastal plants. Gibberellins are a big family of isoprenoid plant hormones, some of which are bioactive growth regulators, controlling seed germination, stem elongation, and flowering [40]. *Gibberella* is a plant growth-promoting fungus producing large amounts of Gibberellins, and was found on the leaves of the detected plant species with relatively high proportions except for the low abundance on CN leaves (0.02% on average). *Periglandula ipomoeae* was a morning-glory symbiotic fungi [41], interesting that a large amount of *Periglandula* detected only on IP leaves (33.1% on average). It might be insufficient to generalize that this genus is IP-specific because we only targeted a small subsection of the plant species diversity of Yongxing Island. However, its distinctive abundance is evidence enough to conclude there is a strong relationship between IP and *Periglandula*, which may be symbiont.

The microorganisms on the surface of leaves are subjected to very stressful environments, including exposure to ultraviolet (UV) radiation [42]. There are also some habitats with high exposure to UV radiation, like Antarctic soil [43] and rock surfaces [44], where the microbiome survived. The ability to tolerate UV radiation is thought to be an important adaptation for the survival of fungi and bacteria in the environment, including the phyllosphere [42,45,46]. Kembel and Mueller [47] concluded that many melanized species belonging to Capnodiales (Dothidiomycetes) and Pleosporales (Dothidiomycetes) commonly could tolerate high UV radiation exposure. It is interesting to see that high abundance of Capnodiales and Pleosporales were detected on the leaves of IP, WC, SS and CN, except for plant SP (Figure S5). Melanized “black yeasts” are also found in the Eurotiomycetes [48], which is dominant in phyllosphere sample SP. This might further generalize a strong relationship between the dominant fungi and the associated host plants. Chemical and physical characteristics of host plant species together with the evolutionary history of the tree could be the reason, but this remains speculative. In another way, UV radiation can also control the growth of fungi [46,49], including pathogenic fungi, which is good for plant health and growth.

### 4.3. Bacterial Communities among Five Plant Species

At the phylum level for bacterial taxonomic composition, the structure of the phyllosphere community in the tropics was similar with that from temperate or tropical plants, including relatively high abundances of Gammaproteobacteria, Alphaproteobacteria, Bacteroidetes, and Actinobacteria, as generally observed from previous studies [17,50,51]. An extremely high abundance of Gammaproteobacteria was found on the leaves of IP and SP with average abundance of 65.2% and 73.7%, respectively. Additionally, Betaproteobacteria, Deltaproteobacteria, and Firmicutes were found on nearly every plant species, indicating that these minor lineages are also consistent residents on leaves and might have significant effects on host plant physiology or ecosystem functioning. Likewise, because our primer set selected to screen out chloroplast DNA, we could not capture any cyanobacteria that may live on the leaves. At the genus level, bacteria belonging to *Pantoea*, *Salinisphaera*, *Pseudomonas*, *Bacteroides*, *Sphingomonas*, *Actinomycetospora*, *Methylobacterium*, *Stenotrophomonas*, *Sphingobacterium*, *Chryseobacterium*, and so on, accounted for a high percentage of the phyllosphere microbial communities (Figure S3). Specially, extremely abundant of *Pantoea* found on IP leaves with the abundance of 59.38%, while it detected on CN leaves with the abundance less than 0.2%. De Maayer [52] concluded that *Pantoea* are frequently isolated from a wide range of ecological niches including soil, water, meat, fish, insects, humans and animals and have various biological roles, as plant epi- or endophytes, biocontrol agents, plant-growth promoters or as pathogens of both plant and animal hosts. *Bacteroides*, predominant intestinal bacteria were enriched in IP and were also able to utilize plant cell wall derived oligosaccharides besides their reported activity toward plant polysaccharides [53]. *Methylobacterium extorquens* expressed proteins that are more abundant on leaves compared with roots, while among the proteins induced during epiphytic growth, enzymes were found to be involved in methanol utilization, prominent stress proteins, and proteins of unknown function [54]. *Salinisphaera*, moderately halophilic bacterium isolated from the brine was reported to encode alkane hydroxylases [55], which probably could degrade alkane. Interestingly, *Salinisphaera* only dominant on CN leaves in our study. The *Cocos nucifera* tree is a member of a group of halophytes plants, which are tolerant of salt water. The mechanism might be that the *Cocos nucifera* tree can route salt to shoots having special compartments within their cells, called vacuoles, which are large storage vats where NaCl can be cordoned off and excluded from the areas on the plant cell where it would do harm [56]. This might provide an appropriate environment for *Salinisphaera*. These unique bacteria were respectively enriched on the leaves of our five detected plants, which might indicate the relationship between the core microorganisms with different plant species. These microorganisms living on the phyllosphere had special traits that help them to adapt the harsh environment degradation [2,4,45], although their ecological functions in the tropical phyllosphere are important for the host plants and the ecosystem need further understood.

Another interesting result is that a large amount of *Alkanindiges* were found only on SS leaves (3.18% on average), and not other plant species (excluding CN with relative abundance less than 0.01%) (Figure S4), which might indicate a strong relationship between SS and *Alkanindiges*. *Alkanindiges* is an alkane-degrading, aerobic coccobacillus, isolated from chronically crude oil-contaminated soil from an oilfield in southern Illinois, which grows very weakly or does not grow in media without hydrocarbons, but its growth is greatly stimulated by straight-chain aliphatic hydrocarbons such as hexadecane and heptadecane and branched aliphatic hydrocarbons such as pristane and squalene [57,58]. Interestingly, SS can produce some chemical constituents, like palmitic acid, phytol and so on [59], which may be the reason why *Alkanindiges* has a unique association with SS leaves.

### 4.4. Diazotrophic Communities among Five Plant Species

At the phylum level for taxonomic composition of diazotrophic community structures detected on the five plant species, the character of the phyllosphere community in the tropics were mainly composed of Proteobacteria, as well as Cyanobacteria, as was generally observed from previous studies [14]. Here, many nitrogen fixing bacteria genera on the leaves, including *Anabaena*, *Dermocarpa*, *Chroococcidiopsis*, *Nostoc*, *Mastigocladus*, *Stanieria*, *Desmonostoc*, and *Trichormus* within Cyanobacteria, and *Rhodoplanes*, *Azospirillum*, *Skermanella*, *Bradyrhizobium*, *Chlorogloeopsis*, and *Methylocaldum* within Alphaproteobacteria, and *Methyloversatilis* within Betaproteobacteria were found among all the detected plant species. Their abundances were different among the five plants, indicating that the proportions of these common nitrogen fixing bacteria are particularly associated with host plant species. *Anabaena* is a cosmopolitan genus showing a wide distribution in diverse aquatic and terrestrial habitats, and represents a rich source of bioactive compounds having industrial and agricultural significance [60]. In our study, a large amount of *Anabaena* were detected on the leaves of SS and SP with the abundance of 16.78% and 14.69%, respectively, next was the IP with the abundance of 2.57%. There are several significant roles of Anabaena, including not only nitrogen fixation, phytohormone-mediated plant growth promotion [61] but also fungicidal activity against species of *Pythium*, *Fusarium* and *Rhizoctonia* [62,63].

Interestingly, a distinctive pattern in the phyllosphere diazotrophic community composition seen in this study is the fact that the genus *Rhodoplanes* (59.58% on average) belonging to Alphaproteobacteria dominates the assemblage on IP phyllosphere sampled here, while a very low abundance of *Rhodoplanes* found in other phyllosphere samples. Whereas members of this group are generally represented in the communities of roots, compared with leaves [64]. Representatives of *Rhodoplanes* have been isolated from pond water [65] and rhizosphere soil, and are characterized by their capacity for growing phototrophically and chemotrophically [66]. Each host plant species in our study has its dominant phyllosphere diazotrophic community, as a distinctive phyllosphere community was detected in tropical trees, and in other plant communities sampled by other groups [17,50]. The reasons why the subgroup of *Rhodoplanes* only dominated in the specific IP phyllosphere among all the detected tropical phyllosphere samples need to be investigated further.

### 4.5. Predictive Metabolism

The *16S rRNA* gene sequences were run through a predictive metagenomics analysis program, revealing that many of the significantly distinct pathways in the different plant-types were associated with amino acid metabolism or transporter proteins (Figure S7, set data 2). About 223 of more than 384 functional pathways (KEGG level 3) were predicted to be significantly different in their abundance per plant-type, and most of these were prevalent in CN (data set 2), which is likely due to its very different microbial community compared to other plant species. The functional pathways that were predicted to be more significant in IP, WC, SS, CN and SP were transporters, ABC transporters, energy metabolism, lipid biosynthesis proteins and secretion system, respectively. The distinct microbial community in the five phyllosphere samples likely explains the predicted significant differential abundance of functional pathways in the phyllosphere samples of five different types of plants. The difference of functional pathways in relative abundance between the five types of phyllosphere samples that we predicted may not be representative of the functional activity of native microorganisms in this habitat. The DNA was extracted from the surface of the plant leaves, which does not necessarily mean that the organisms are native and metabolically active in this habitat, once this part of the plants is exposed to the rain, wind, and so it is possible that non-autochthonous microorganisms were deposited on the leaf surfaces from other habitats. In addition, the limitations of molecular approach would affect the differences between phyllosphere microorganisms of different plant species, for example, the differences of morphology and physiology of different plant species would affect the extraction efficiency of DNA. In future studies, we still need to develop advanced methods to reduce the interference of non-autochthonous microorganisms or the differences of morphology and physiology of different plant species, so as to more accurately characterize the diversity of phyllosphere epiphytes and their relationship with plants.

## 5. Conclusions

In conclusion, the abundance, diversity and composition of the phyllospheric microbial communities differed greatly among the five plants. There was a small proportion of shared microorganisms among the five different plants, while unique fungi, bacteria and diazotrophs were enriched for different host plant species in this study. Some of the most abundant organisms found in the communities of these different host plants were involved in important biogeochemical cycles that can benefit their host, including carbon and nitrogen cycles, while some microorganisms are potential pathogens, which need us to pay more attention to the health of these coastal plants.

## Figures and Tables

**Table 1:** Alpha-diversity of microbes between different phyllosphere samples.

Taxa	Samples	Shannon	Chao1	Heip	Coverage
Fungi	IP	2.39 ± 0.07c	279 ± 16b	0.050 ± 0.001b	0.9988 ± 0.0001a
	WC	1.35 ± 0.14b	290 ± 21b	0.013 ± 0.003a	0.9989 ± 0.0000a
	SS	3.22 ± 0.15d	483 ± 3c	0.057 ± 0.005c	0.9985 ± 0.0003a
	CN	1.33 ± 0.02b	278 ± 41b	0.014 ± 0.001a	0.9988 ± 0.0002a
	SP	0.77 ± 0.15a	146 ± 44a	0.013 ± 0.001a	0.9994 ± 0.0003b
Bacteria	IP	2.13 ± 0.05a	498 ± 37a	0.023 ± 0.001a	0.9968 ± 0.0008b
	WC	4.40 ± 0.02d	1288 ± 80c	0.090 ± 0.010c	0.9911 ± 0.0025ab
	SS	5.52 ± 0.04e	2070 ± 55e	0.149 ± 0.011d	0.9862 ± 0.0030a
	CN	3.87 ± 0.08c	1549 ± 223d	0.046 ± 0.005b	0.9865 ± 0.0036a
	SP	2.78 ± 0.07b	1034 ± 122b	0.024 ± 0.003a	0.9927 ± 0.0016b
Diazotrophs	IP	1.87 ± 0.20a	137 ± 16a	0.045 ± 0.005a	0.9989 ± 0.0003a
	WC	4.30 ± 0.02c	219 ± 8b	0.346 ± 0.013d	0.9992 ± 0.0004a
	SS	3.51 ± 0.25b	192 ± 30b	0.183 ± 0.053b	0.9992 ± 0.0002a
	CN	4.73 ± 0.07d	467 ± 46d	0.253 ± 0.044c	0.9980 ± 0.0006a
	SP	4.33 ± 0.21c	279 ± 29c	0.285 ± 0.040cd	0.9985 ± 0.0008a

Different letters between five phyllosphere indicate significant differences among samples at *p* < 0.05.

## Supplementary Materials

The following are available online at https://www.mdpi.com/2076-2607/7/11/525/s1, Figure S1: Relative abundance of fungi on phylum level of all the phyllosphere samples, Figure S2: The relative abundances of shared fungi at genus level between five phyllosphere samples, Figure S3: The relative abundances of shared bacteria at genus level between five phyllosphere samples, Figure S4: Heatmap of bacteria on genus level with relative abundance of the taxa comprising more than 1.0% of the bacterial sequences in each sample between five phyllosphere samples, Figure S5, Relative abundance of fungi on order level of all the phyllosphere samples, Figure S6 Relative abundance of 45 level 2 Kyoto Encyclopedia of Genes and Genomes (KEGG) orthology groups (KOs) represented in data set between five plant species, Figure S7 Heatmap of relative abundance of the top 50 functional pathways (KEGG level 3) in five different types of phyllosphere samples, Table S1: Primers used in this study, Table S2: Molecular detection of fungi, bacteria, and diazotrophs, Table S3: Analysis of similarities (ANOSIM) of fungal communities between the samples, Table S4: Analysis of similarities (ANOSIM) of bacterial communities between the samples, Table S5: Analysis of similarities (ANOSIM) of diazotrophic communities between the samples.

## References

[1] Lindow S.E., Leveau J.H. (2002). Phyllosphere microbiology. Curr. Opin. Biotechnol..

[2] Beattie G.A., Lindow S.E. (1999). Bacterial colonization of leaves: A spectrum of strategies. Phytopathology.

[3] Kishore G.K., Pande S., Podile A.R. (2005). Biological control of late leaf spot of peanut (arachis hypogaea) with chitinolytic bacteria. Phytopathology.

[4] Lindow S.E., Brandl M.T. (2003). Microbiology of the phyllosphere. Appl. Environ. Microbiol..

[5] Terhonen E., Blumenstein K., Kovalchuk A., Asiegbu F.O. (2019). Forest tree microbiomes and associated fungal endophytes: Functional roles and impact on forest health. Forests.

[6] Porras-Alfaro A., Bayman P., VanAlfen N.K., Bruening G., Leach J.E. (2011). Hidden fungi, emergent properties: Endophytes and microbiomes. Annual Review of Phytopathology.

[7] Innerebner G., Knief C., Vorholt J.A. (2011). Protection of arabidopsis thaliana against leaf-pathogenic pseudomonas syringae by sphingomonas strains in a controlled model system. Appl. Environ. Microbiol..

[8] Lugtenberg B.J.J., de Weger L.A., Bennett J.W. (1991). Microbial stimulation of plant growth and protection from disease. Curr. Opin. Biotechnol..

[9] Kinkel L.L., Wilson M., Lindow S.E. (2000). Plant species and plant incubation conditions influence variability in epiphytic bacterial population size. Microb. Ecol..

[10] Hardoim P.R., van Overbeek L.S., Berg G., Pirttila A.M., Compant S., Campisano A., Doring M., Sessitsch A. (2015). The hidden world within plants: Ecological and evolutionary considerations for defining functioning of microbial endophytes. Microbiol. Mol. Biol. Rev..

[11] Dulla G., Marco M., Quinones B., Lindow S. (2005). A closer look at pseudomonas syringae as a leaf colonist- the pathogen p-syringae thrives on healthy plants by employing quorum sensing, virulence factors, and other traits. Asm News.

[12] Kovalchuk A., Mukrimin M., Zeng Z., Raffaello T., Liu M.X., Kasanen R., Sun H., Asiegbu F.O. (2018). Mycobiome analysis of asymptomatic and symptomatic norway spruce trees naturally infected by the conifer pathogens *Heterobasidion* spp.. Environ. Microbiol. Rep..

[13] Papen H., Geβler A., Zumbusch E., Rennenberg H. (2002). Chemolithoautotrophic nitrifiers in the phyllosphere of a spruce ecosystem receiving high atmospheric nitrogen input. Curr. Microbiol..

[14] Fuernkranz M., Wanek W., Richter A., Abell G., Rasche F., Sessitsch A. (2008). Nitrogen fixation by phyllosphere bacteria associated with higher plants and their colonizing epiphytes of a tropical lowland rainforest of costa rica. ISME J..

[15] Van Aken B., Peres C.M., Doty S.L., Yoon J.M., Schnoor J.L. (2004). Methylobacterium populi sp nov., a novel aerobic, pink-pigmented, facultatively methylotrophic, methane-utilizing bacterium isolated from poplar trees (populus deltoides x nigra dn34). Int. J. Syst. Evol. Microbiol..

[16] Baldrian P. (2016). Forest microbiome: Diversity, complexity and dynamics. Fems Microbiol. Rev..

[17] Redford A.J., Bowers R.M., Knight R., Linhart Y., Fierer N. (2010). The ecology of the phyllosphere: Geographic and phylogenetic variability in the distribution of bacteria on tree leaves. Environ. Microbiol..

[18] Kembel S.W., O’Connor T.K., Arnold H.K., Hubbell S.P., Wright S.J., Green J.L. (2014). Relationships between phyllosphere bacterial communities and plant functional traits in a neotropical forest. Proc. Natl. Acad. Sci. USA.

[19] Jumpponen A., Jones K.L. (2009). Massively parallel 454 sequencing indicates hyperdiverse fungal communities in temperate quercus macrocarpa phyllosphere. New Phytol..

[20] Abril A.B., Torres P.A., Bucher E.H. (2005). The importance of phyllosphere microbial populations in nitrogen cycling in the chaco semi-arid woodland. J. Trop. Ecol..

[21] Cleveland C.C., Townsend A.R., Schimel D.S., Fisher H., Howarth R.W., Hedin L.O., Perakis S.S., Latty E.F., Fischer J.C.V., Elseroad A. (1999). Global patterns of terrestrial biological nitrogen (n2) fixation in natural ecosystems. Glob. Biogeochem. Cycles.

[22] Roggy J.C., Prevost M.F., Garbaye J., Domenach A.M. (1999). Nitrogen cycling in the tropical rain forest of french guiana: Comparison of two sites with contrasting soil types using delta n-15. J. Trop. Ecol..

[23] Poly F., Monrozier L.J., Bally R. (2001). Improvement in the rflp procedure for studying the diversity of nifh genes in communities of nitrogen fixers in soil. Res. Microbiol..

[24] Segata N., Izard J., Waldron L., Gevers D., Miropolsky L., Garrett W.S., Huttenhower C. (2011). Metagenomic biomarker discovery and explanation. Genome Biol..

[25] Asshauer K.P., Wemheuer B., Daniel R., Meinicke P. (2015). Tax4fun: Predicting functional profiles from metagenomic 16s rrna data. Bioinformatics.

[26] Rea M.C., Dobson A., O’Sullivan O., Crispie F., Fouhy F., Cotter P.D., Shanahan F., Kiely B., Hill C., Ross R.P. (2011). Effect of broad- and narrow-spectrum antimicrobials on clostridium difficile and microbial diversity in a model of the distal colon. Proc. Natl. Acad. Sci. USA.

[27] Heip C. (1974). A new index measuring evenness. J. Mar. Biol. Assoc. UK.

[28] Gotelli N.J., Colwell R.K. (2011). Estimating species richness. Biol. Divers. Front. Meas. Assess..

[29] Barka E.A., Vatsa P., Sanchez L., Gaveau-Vaillant N., Jacquard C., Klenk H.P., Clement C., Ouhdouch Y., van Wezel G.P. (2016). Cd taxonomy, physiology, and natural products of actinobacteria. Microbiol. Mol. Biol. Rev..

[30] Ellison J.C., Botero C.M., Cervantes O., Finkl C.W. (2018). Pacific island beaches: Values, threats and rehabilitation. Beach Management Tools-Concepts, Methodologies and Case Studies.

[31] Lindow S.E., Andersen G.L. (1996). Influence of immigration on epiphytic bacterial populations on navel orange leaves. Appl. Environ. Microbiol..

[32] Yadav R.K.P., Karamanoli K., Vokou D. (2005). Bacterial colonization of the phyllosphere of mediterranean perennial species as influenced by leaf structural and chemical features. Microb. Ecol..

[33] Bloom A.J., Chapin F.S., Mooney H.A. (1985). Resource limitation in plants-an economic analogy. Annu. Rev. Ecol. Syst..

[34] Ruppel S., Krumbein A., Schreiner M. (2008). Composition of the phyllospheric microbial populations on vegetable plants with different glucosinolate and carotenoid compositions. Microb. Ecol..

[35] Yao H., Sun X., He C., Maitra P., Li X.-C., Guo L.-D. (2019). Phyllosphere epiphytic and endophytic fungal community and network structures differ in a tropical mangrove ecosystem. Microbiome.

[36] Rodriguez R.J., White J.F., Arnold A.E., Redman R.S. (2009). Fungal endophytes: Diversity and functional roles. New Phytol..

[37] Shah A., Hassan Q.P., Mushtaq S., Shah A.M., Hussain A. (2017). Chemoprofile and functional diversity of fungal and bacterial endophytes and role of ecofactors—A review. J. Basic Microbiol..

[38] Osono T., Mori A. (2003). Colonization of japanese beech leaves by phyllosphere fungi. Mycoscience.

[39] Kirk P.M., Cannon P.F., Minter D.W., Stalpers J.A. (2008). Dictionary of the Fungi.

[40] Tudzynski B. (1999). Biosynthesis of gibberellins in gibberella fujikuroi: Biomolecular aspects. Appl. Microbiol. Biotechnol..

[41] Schardl C.L., Young C.A., Hesse U., Amyotte S.G., Andreeva K., Calie P.J., Fleetwood D.J., Haws D.C., Moore N., Oeser B. (2013). Plant-symbiotic fungi as chemical engineers: Multi-genome analysis of the clavicipitaceae reveals dynamics of alkaloid loci. Plos Genet..

[42] Kadivar H., Stapleton A.E. (2003). Ultraviolet radiation alters maize phyllosphere bacterial diversity. Microb. Ecol..

[43] Tosi S., Onofri S., Brusoni M., Zucconi L., Vishniac H. (2005). Response of antarctic soil fungal assemblages to experimental warming and reduction of uv radiation. Polar Biol..

[44] Ruibal C., Gueidan C., Selbmann L., Gorbushina A.A., Crous P.W., Groenewald J.Z., Muggia L., Grube M., Isola D., Schoch C.L. (2009). Phylogeny of rock-inhabiting fungi related to dothideomycetes. Stud. Mycol..

[45] Sundin G.W., Jacobs J.L. (1999). Ultraviolet radiation (uvr) sensitivity analysis and uvr survival strategies of a bacterial community from the phyllosphere of field-grown peanut (arachis hypogeae l.). Microb. Ecol..

[46] Hughes K.A., Lawley B., Newsham K.K. (2003). Solar uv-b radiation inhibits the growth of antarctic terrestrial fungi. Appl. Environ. Microbiol..

[47] Kembel S.W., Mueller R.C. (2014). Plant traits and taxonomy drive host associations in tropical phyllosphere fungal communities. Botany.

[48] Geiser D.M., Gueidan C., Miadlikowska J., Lutzoni F., Kauff F., Hofstetter V., Fraker E., Schoch C.L., Tibell L., Untereiner W.A. (2006). Eurotiomycetes: Eurotiomycetidae and chaetothyriomycetidae. Mycologia.

[49] Ensminger P.A. (1993). Control of development in plants and fungi by far-uv radiation. Physiol. Plant..

[50] Kim M., Singh D., Lai-Hoe A., Go R., Abdul Rahim R., Ainuddin A.N., Chun J., Adams J.M. (2012). Distinctive phyllosphere bacterial communities in tropical trees. Microb. Ecol..

[51] Ugarelli K., Laas P., Stingl U. (2019). The microbial communities of leaves and roots associated with turtle grass (thalassia testudinum) and manatee grass (syringodium filliforme) are distinct from seawater and sediment communities, but are similar between species and sampling sites. Microorganisms.

[52] De Maayer P., Chan W.Y., Blom J., Venter S.N., Duffy B., Smits T.H.M., Coutinho T.A. (2012). The large universal pantoea plasmid lpp-1 plays a major role in biological and ecological diversification. BMC Genom..

[53] Van Laere K.M.J., Hartemink R., Bosveld M., Schols H.A., Voragen A.G.J. (2000). Fermentation of plant cell wall derived polysaccharides and their corresponding oligosaccharides by intestinal bacteria. J. Agric. Food Chem..

[54] Gourion B., Rossignol M., Vorholt J.A. (2006). A proteomic study of methylobacterium extorquens reveals a response regulator essential for epiphytic growth. Proc. Natl. Acad. Sci. USA.

[55] Liping W., Wanpeng W., Qiliang L., Zongze S. (2010). Gene diversity of cyp153a and alkb alkane hydroxylases in oil-degrading bacteria isolated from the atlantic ocean. Environ. Microbiol..

[56] Shannon M.C. (1994). The Effects of Salinity on Cellular and Biochemical Processes Associated with Salt Tolerance in Tropical Plants.

[57] Woo P.C.Y., Tse H., Lau S.K.P., Leung K.-W., Woo G.K.S., Wong M.K.M., Ho C.-M., Yuen K.-Y. (2005). Alkanindiges hongkongensis sp. nov. A novel alkanindiges species isolated from a patient with parotid abscess. Syst. Appl. Microbiol..

[58] Bogan B.W., Sullivan W.R., Kayser K.J., Derr K.D., Aldrich H.C., Peterek J.R. (2003). Alkanindiges illinoisensis gen. Nov., sp. nov., an obligately hydrocarbonoclastic, aerobic squalane-degrading bacterium isolated from oilfield soils. Int. J. Syst. Evol. Microbiol..

[59] Li M., Lou H.X., Huang C.S., Lu Q. (2015). Main volatile chemical constituents and antimicrobial activity of scaevola sericea leaves. Technol. Dev. Chem. Ind..

[60] Prasanna R., Gupta V., Natarajan C., Chaudhary V. (2010). Bioprospecting for genes involved in the production of chitosanases and microcystin-like compounds in anabaena strains. World J. Microbiol. Biotechnol..

[61] Prasanna R., Joshi M., Rana A., Nain L. (2010). Modulation of iaa production in cyanobacteria by tryptophan and light. Pol. J. Microbiol..

[62] Chaudhary V., Prasanna R., Bhatnagar A.K. (2012). Modulation of fungicidal potential of anabaena strains by light and temperature. Folia Microbiol..

[63] Prasanna R., Chaudhary V., Gupta V., Babu S., Kumar A., Singh R., Shivay Y.S., Nain L. (2013). Cyanobacteria mediated plant growth promotion and bioprotection against fusarium wilt in tomato. Eur. J. Plant Pathol..

[64] Santana R.S.M., Fernandes G.W., Avila M.P., Reis M.P., de Araujo F.M.G., Salim A.C.M., Oliveira G., Chartone-Souza E., Nascimento A.M.A. (2016). Endophytic microbiota associated with the root tips and leaves of baccharis dracunculifolia. Braz. Arch. Biol. Technol..

[65] Chakravarthy S.K., Ramaprasad E.V.V., Shobha E., Sasikala C., Ramana C.V. (2012). Rhodoplanes piscinae sp nov isolated from pond water. Int. J. Syst. Evol. Microbiol..

[66] Srinivas A., Sasikala C., Ramana C.V. (2014). Rhodoplanes oryzae sp. nov., a phototrophic alphaproteobacterium isolated from the rhizosphere soil of paddy. Int. J. Syst. Evol. Microbiol..

