# Erratum: Bao, L.; Cai, W.; Zhang, X.; Liu, J.; Chen, H.; Wei, Y.; Jia, X.; Bai, Z. Distinct Microbial Community of Phyllosphere Associated with Five Tropical Plants on Yongxing Island, South China Sea. *Microorganisms* 2019, *7*, 525

**DOI:** 10.3390/microorganisms8040570

**Published:** 2020-04-15

**Authors:** Lijun Bao, Wenyang Cai, Xiaofen Zhang, Jinhong Liu, Hao Chen, Yuansong Wei, Xiuxiu Jia, Zhihui Bai

**Affiliations:** 1 Research Center for Eco-Environmental Sciences, Chinese Academy of Sciences, Beijing 100085, China; ljbao_st@rcees.ac.cn (L.B.); dretfr456@gmail.com (W.C.); chenhao@rcees.ac.cn (H.C.); yswei@rcees.ac.cn (Y.W.); 2 College of Resources and Environment, University of Chinese Academy of Sciences, Beijing 100049, China; 3 College of Biological Sciences and Biotechnology, Beijing Forestry University, Beijing, 100083, China; 4 Institute of Naval Engineering Design & Research, Beijing 100070, China; fenny425@126.com (X.Z.); liujh690@sina.com (J.L.); 5 School of Environmental Science and Engineering, Hebei University of Science and Technology, Shijiazhuang 050018, China

The authors wish to make the following erratum in this paper [1].

There are mistakes in the spelling of the first and second institute names of the authors:

1. **Research Center for Eco-Environment Sciences, Chinese Academy of Sciences, Beijing 100085, China; ljbao_st@rcees.ac.cn (L.B.); dretfr456@gmail.com (W.C.); chenhao@rcees.ac.cn (H.C.); yswei@rcees.ac.cn (Y.W.)**
2. **College of Resources and Environment, University of Chinese Academy of Science, Beijing, 100049, China**

The corrected spelling should be:

1. **Research Center for Eco-Environmental Sciences, Chinese Academy of Sciences, Beijing 100085, China; ljbao_st@rcees.ac.cn (L.B.); dretfr456@gmail.com (W.C.); chenhao@rcees.ac.cn (H.C.); yswei@rcees.ac.cn (Y.W.)**
2. **College of Resources and Environment, University of Chinese Academy of Sciences, Beijing, 100049, China**

The authors apologize for any inconvenience this may have caused.
