# Supplementary material for: Distinct Microbial Community of Phyllosphere Associated with Five Tropical Plants on Yongxing Island, South China Sea
Source: Microorganisms. 2019 Nov 4;7(11):525. doi: 10.3390/microorganisms7110525 (PMC6920945; doi:10.3390/microorganisms7110525)
Supplement: Supplementary file 1 [file microorganisms-07-00525-s001.zip › microorganisims-603163 supplementary/Table S1-S5.docx]

**Table S1.** Primers used in this study.

| **Primer name** | **Primer sequence (5’-3’)** | **Target gene** | **Annealing T °C** |
| --- | --- | --- | --- |
| ITS1 | CTTGGTCATTTAGAGGAAGTAA | ITS1 region | 55°C |
| ITS2 | GCTGCGTTCTTCATCGATGC |  |  |
| 799F | AACMGGATTAGATACCCKG | bacteria *16S rRNA* gene | 57°C |
| 1115R | AGGGTTGCGCTCGTTG |  |  |
| polF | TGCGAYCCSAARGCBGACTC | *nifH* gene | 58°C |
| polR | ATSGCCATCATYTCRCCGGA |  |  |

**Table S2.** Molecular detection of fungi, bacteria, and diazotrophs.

| **Sample ID** | **Samples** | **Number of OTUs** | | |
| --- | --- | --- | --- | --- |
|  |  | Fungi | Bacteria | Diazotrophs |
| IP1 | *Ipomoea pes-caprae* | 186 | 314 | 140 |
| IP2 |  | 212 | 304 | 104 |
| IP3 |  | 201 | 359 | 127 |
| WC1 | *Wedelia chinensis* | 216 | 952 | 217 |
| WC2 |  | 204 | 961 | 206 |
| WC3 |  | 239 | 801 | 212 |
| SS1 | *Scaevola sericea* | 393 | 1780 | 163 |
| SS2 |  | 431 | 1584 | 179 |
| SS3 |  | 448 | 1637 | 209 |
| CN1 | *Cocos nucifera* | 195 | 898 | 480 |
| CN2 |  | 216 | 980 | 470 |
| CN3 |  | 189 | 1234 | 396 |
| SP1 | *Sesuvium portulacastrum* | 71 | 546 | 303 |
| SP2 |  | 129 | 697 | 252 |
| SP3 |  | 85 | 680 | 249 |

**Table S3.** Analysis of similarities (ANOSIM) of fungal communities between the samples.

|  | ***R*** | ***p*** |
| --- | --- | --- |
| IP vs WC vs SS vs CN vs SP | **1** | **0.001** |
| WC, CN vs SP | **1** | **0.007** |
| IP, SS vs SP | **1** | **0.017** |
| IP vs SS | 1 | 0.106 |
| WC vs CN | 1 | 0.097 |

Values in bold indicate significant differences at p < 0.01/ p < 0.05.

**Table S4.** Analysis of similarities (ANOSIM) of bacterial communities between the samples.

|  | ***R*** | ***p*** |
| --- | --- | --- |
| IP vs WC vs SS vs CN vs SP | **1** | **0.001** |
| WC, SS, CN vs IP, SP | **0.8235** | **0.001** |
| WC, SS vs CN | **0.9985** | **0.001** |
| WC vs SS | 1 | 0.095 |
| IP vs SP | 1 | 0.096 |

Values in bold indicate significant differences at p < 0.01/ p < 0.05.

**Table S5.** Analysis of similarities (ANOSIM) of diazotrophic communities between the samples.

|  | ***R*** | ***p*** |
| --- | --- | --- |
| IP vs WC vs SS vs CN vs SP | **1** | **0.001** |
| WC, CN, SP vs IP, SS | **0.7473** | **0.001** |
| CN, SP vs WC | **1** | **0.01** |
| CN vs SP | 1 | 0.094 |
| IP vs SS | 1 | 0.102 |

Values in bold indicate significant differences at p < 0.01/ p < 0.05.
