# Supplementary figures and images for: Distinct Microbial Community of Phyllosphere Associated with Five Tropical Plants on Yongxing Island, South China Sea
Source: Microorganisms. 2019 Nov 4;7(11):525. doi: 10.3390/microorganisms7110525 (PMC6920945; doi:10.3390/microorganisms7110525)

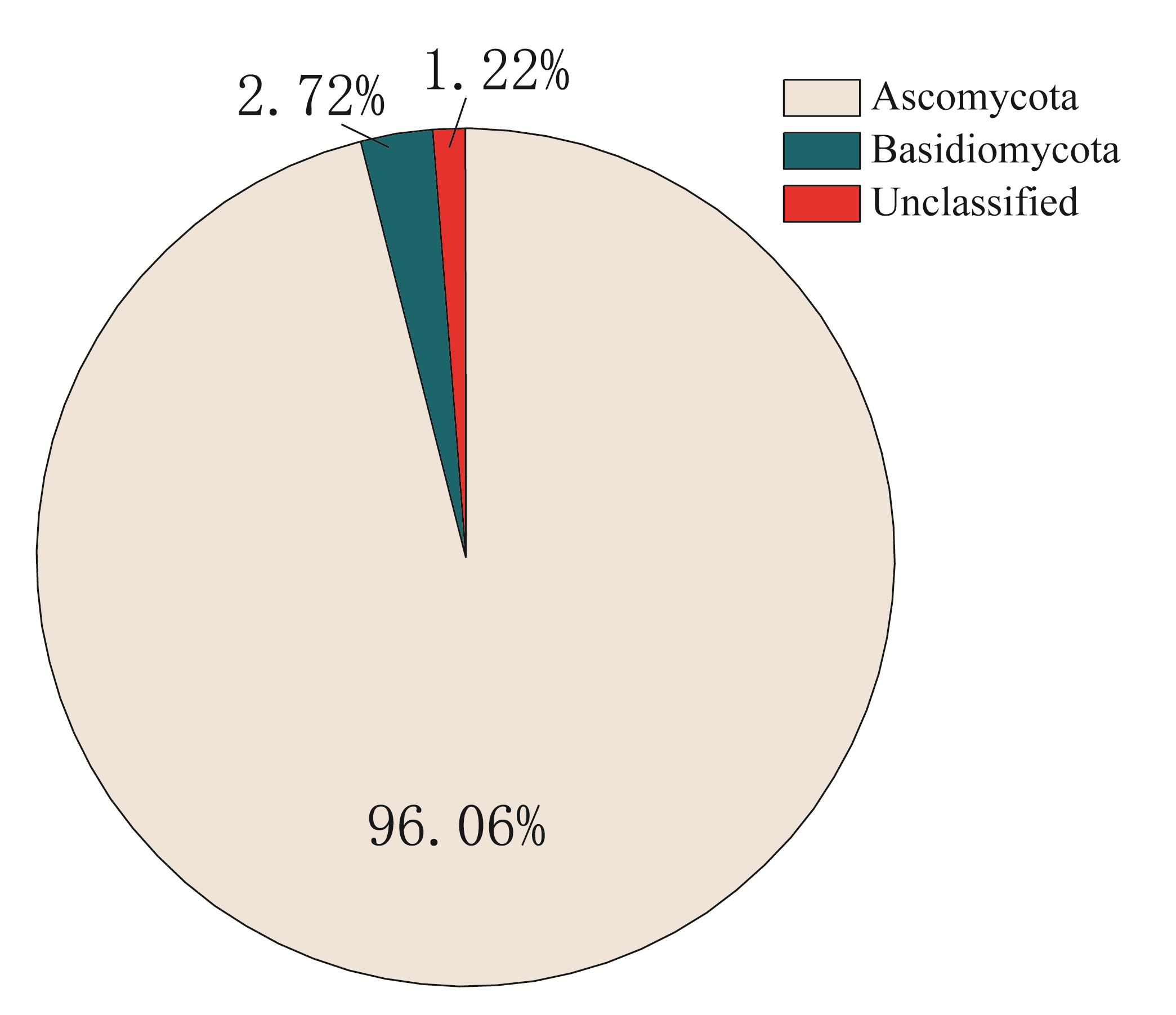

Supplement: Supplementary file 1 [file microorganisms-07-00525-s001.zip › microorganisims-603163 supplementary/Figure S1.tif]

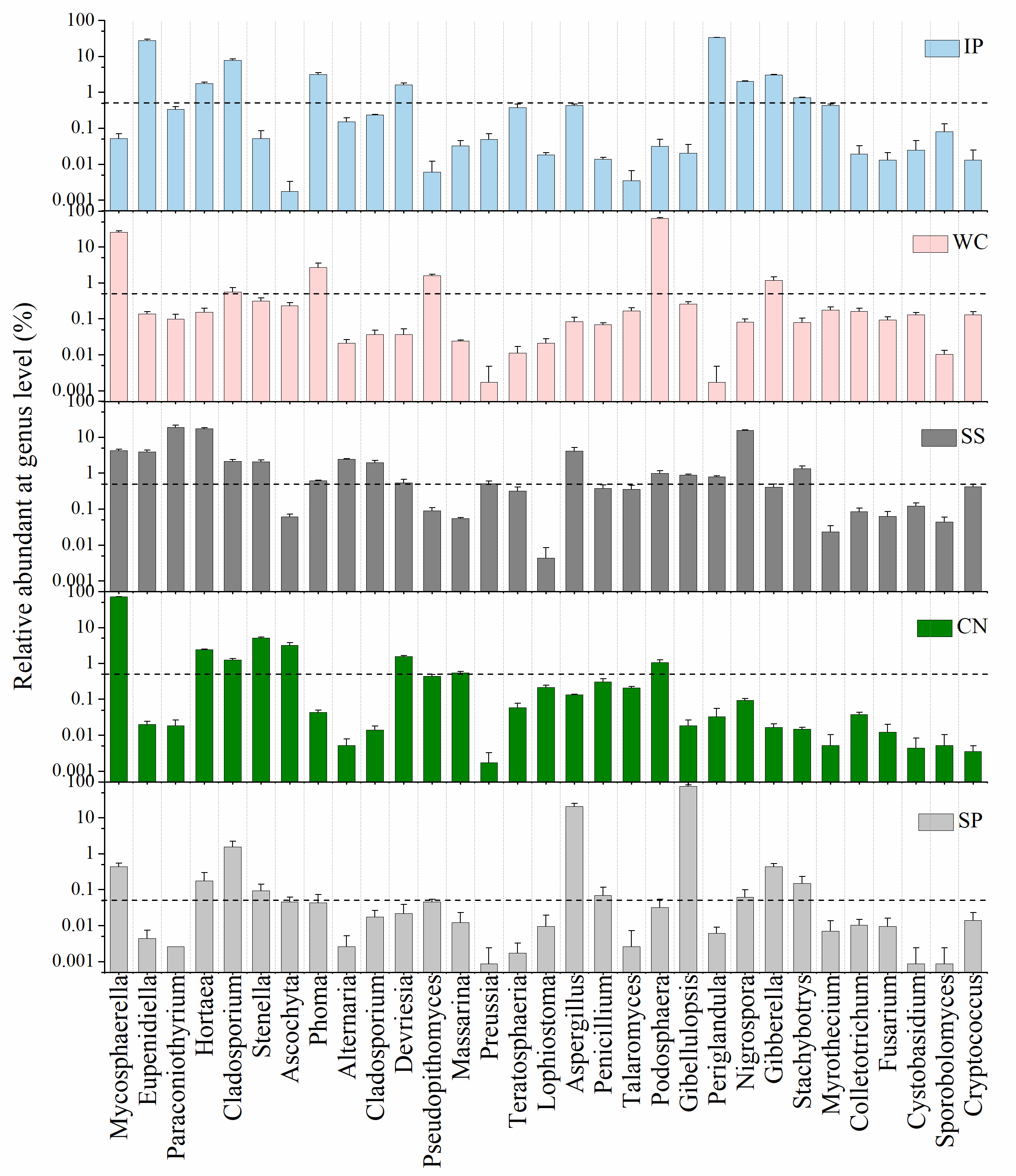

Supplement: Supplementary file 1 [file microorganisms-07-00525-s001.zip › microorganisims-603163 supplementary/Figure S2.tif]

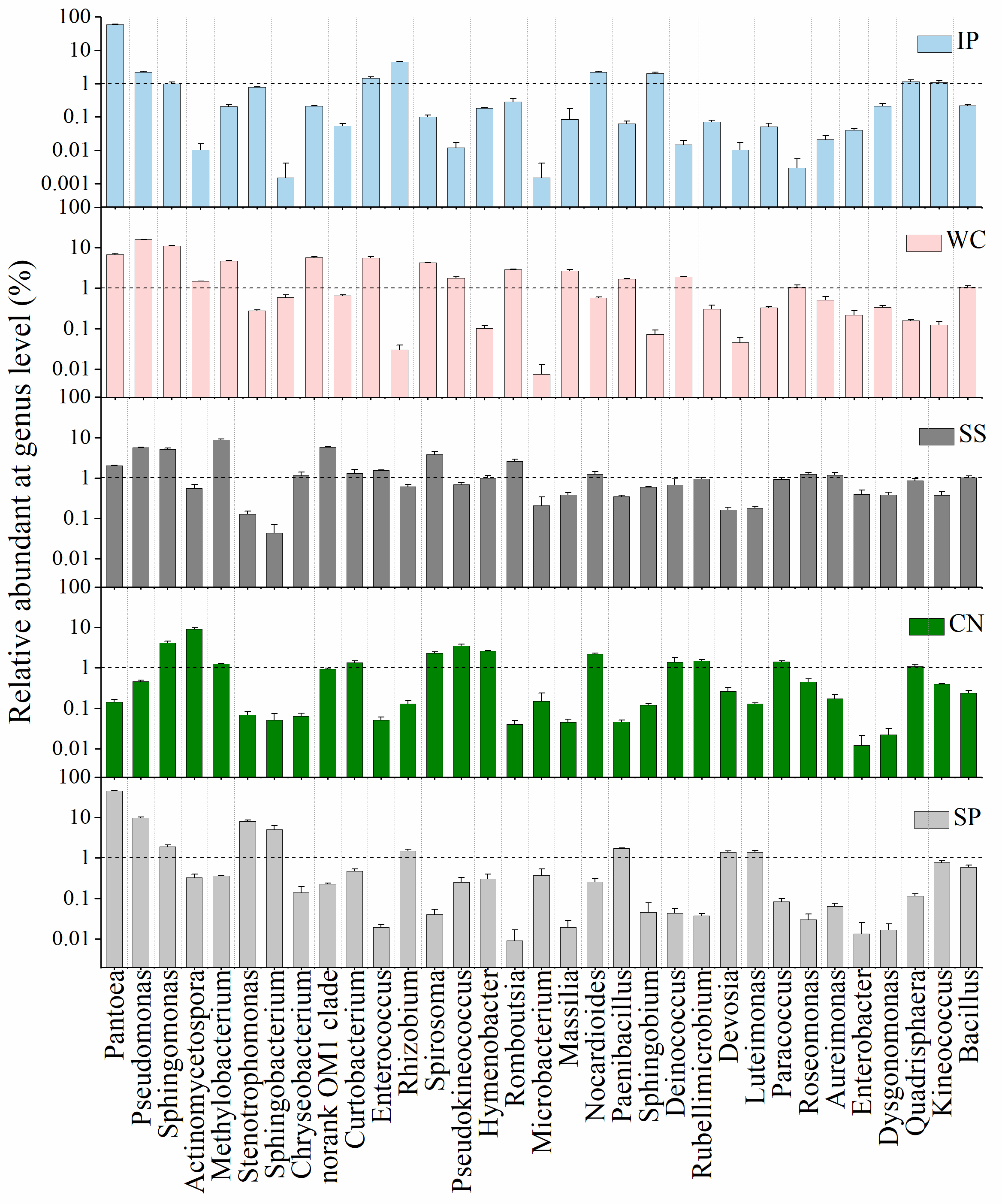

Supplement: Supplementary file 1 [file microorganisms-07-00525-s001.zip › microorganisims-603163 supplementary/Figure S3.tif]

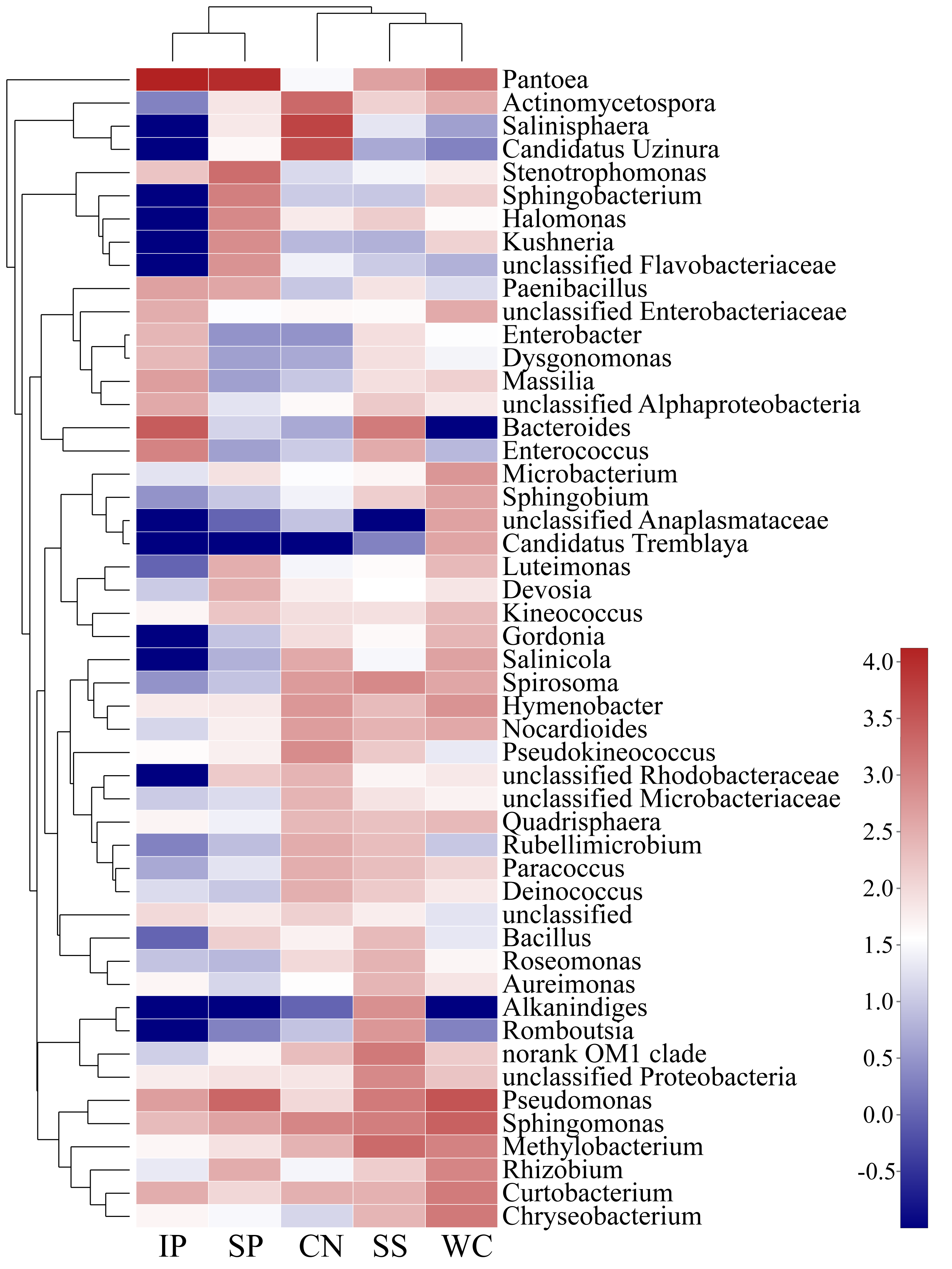

Supplement: Supplementary file 1 [file microorganisms-07-00525-s001.zip › microorganisims-603163 supplementary/Figure S4.tif]

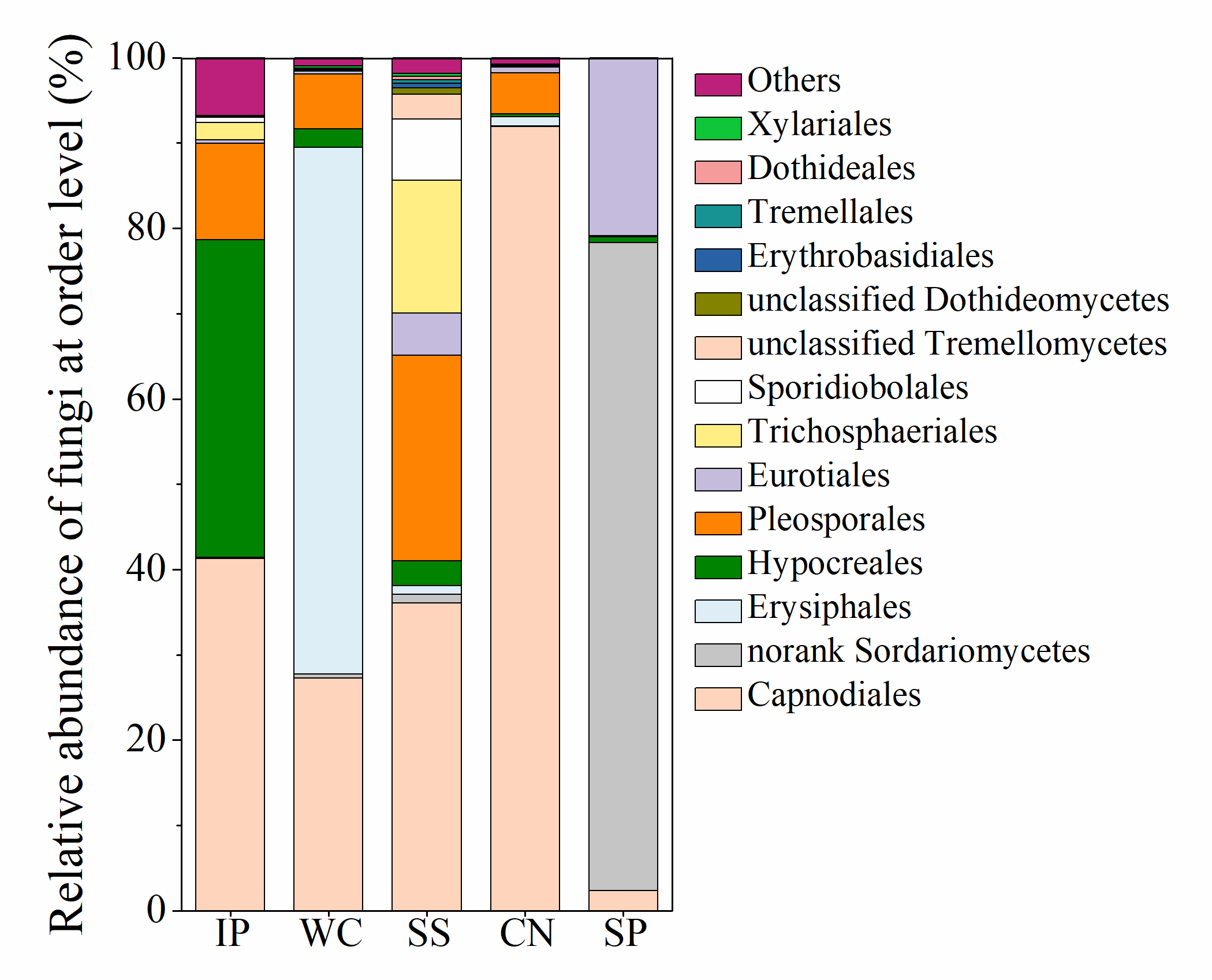

Supplement: Supplementary file 1 [file microorganisms-07-00525-s001.zip › microorganisims-603163 supplementary/Figures S5.tif]

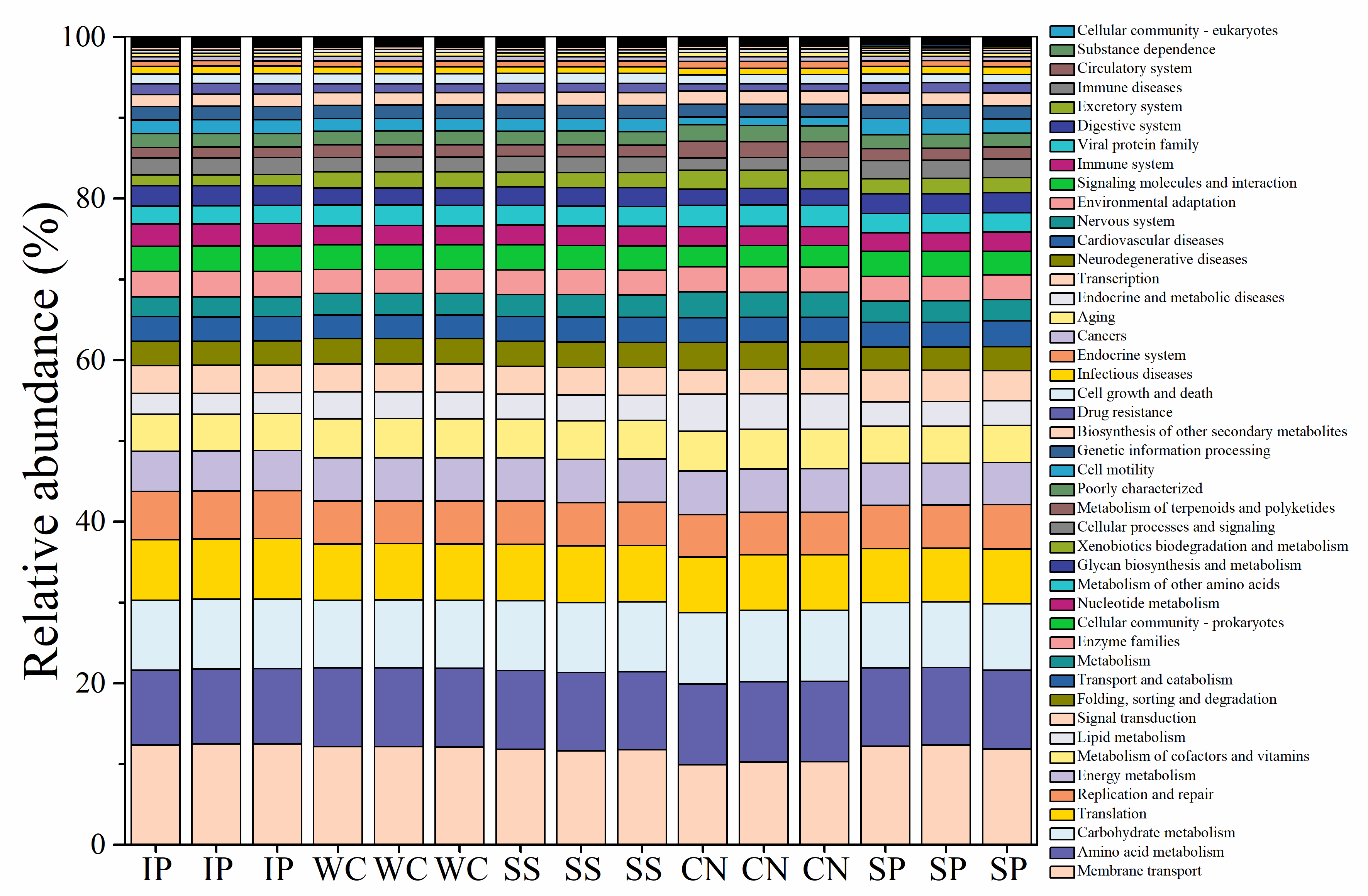

Supplement: Supplementary file 1 [file microorganisms-07-00525-s001.zip › microorganisims-603163 supplementary/Figures S6.tif]

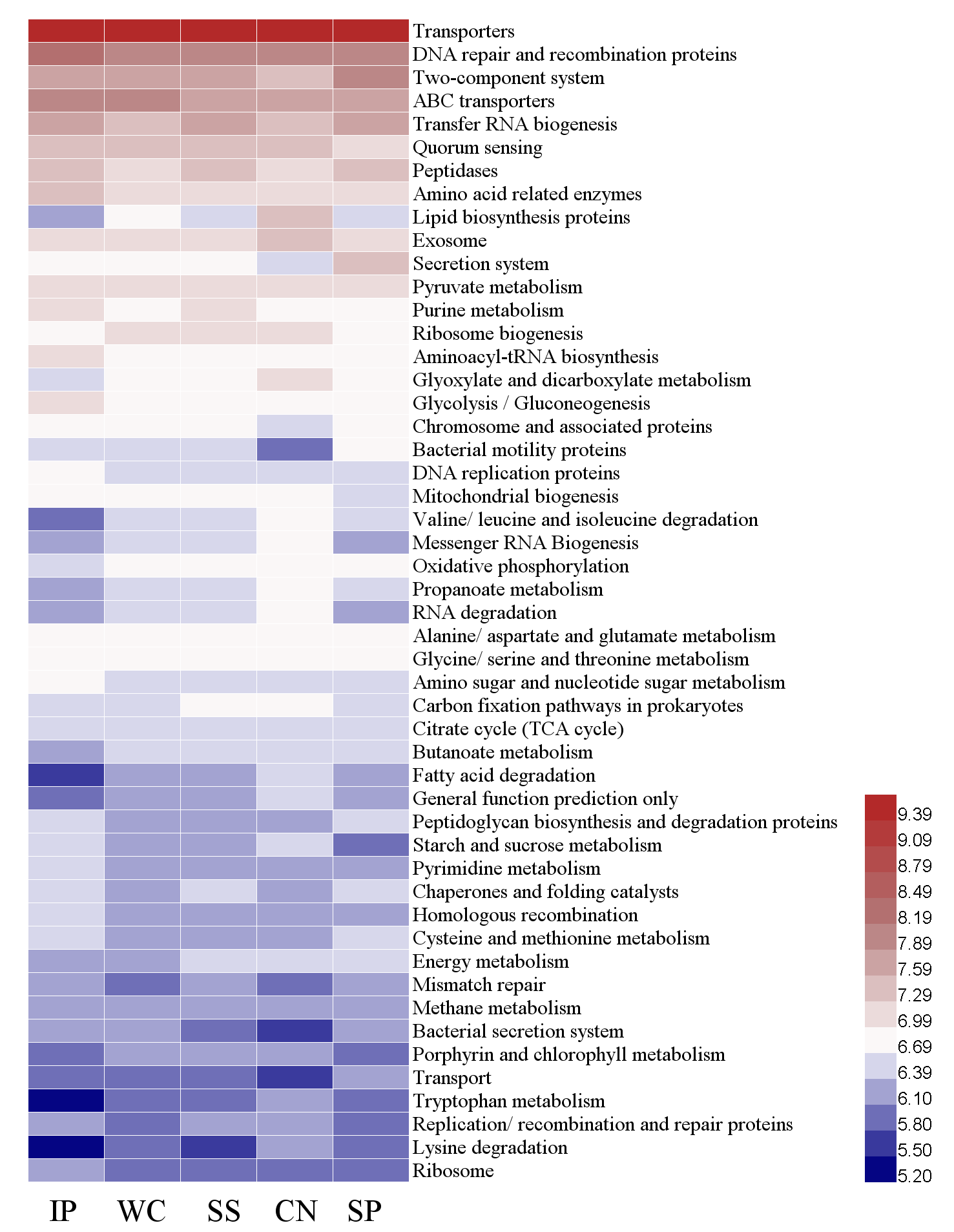

Supplement: Supplementary file 1 [file microorganisms-07-00525-s001.zip › microorganisims-603163 supplementary/Figures S7.tiff]
